# The avian influenza A virus receptor SA-α2,3-Gal is expressed in the porcine nasal mucosa sustaining the pig as a mixing vessel for new influenza viruses

**DOI:** 10.1016/j.virusres.2023.199304

**Published:** 2024-01-03

**Authors:** Charlotte Kristensen, Lars E. Larsen, Ramona Trebbien, Henrik E. Jensen

**Affiliations:** a Department of Veterinary and Animal Sciences, University of Copenhagen, Frederiksberg C, Denmark; b Department of Virus and Microbiological Special Diagnostics, Statens Serum Institut, Copenhagen S, Denmark

**Keywords:** Influenza A virus, Host tropism, Sialic acid, Experimental infections, Receptor, Pigs

## Abstract

Influenza A viruses (IAVs) originate from wild birds but have on several occasions jumped host barriers and are now also circulating in humans and mammals. The IAV host receptors (glycans with galactose linked to a sialic acid (SA) in an α2,3 or α2,6 linkage) are crucial host factors restricting inter-species transmission. In general, avian-origin IAVs show a preference for SA-α2,3 (avian receptor), whereas IAVs isolated from humans and pigs prefer SA-α2,6 (human receptor). N-acetylneuraminic acid (Neu5Ac) and N-glycolylneuraminic acid (Neu5Gc) are the two major SAs. Neu5Ac is expressed in all species, whereas Neu5Gc is only expressed in a limited number of domestic species such as pigs and horses, but not in humans. Despite that previous studies have shown that the IAV host receptor distribution appears to be similar in pigs and humans, none of these studies have investigated the expression of Neu5Gc-α2,6 *in situ* in porcine tissues. Thus, the aim of this study was to elucidate the distribution of IAV host receptors expressed in the porcine respiratory tract and relate the expression to the viral tropism of diverse host-adapted IAVs.

The IAV receptor (SA-α2,3 and SA-α2,6) distribution and the presence of specifically Neu5Gc-α2,6 in the porcine nasal, tracheal, and lung tissues was investigated by lectin histochemistry. Furthermore, IAV immunohistochemistry was performed on tissues from pigs experimentally infected with IAVs, either adapted to pigs or humans, to investigate the significance of the IAV host receptors and the tropism of the diverse host-adapted IAVs. We document for the first time the expression of the avian receptor on the surface of the porcine nasal mucosa and an equal expression of Neu5Ac-α2,6 and Neu5Gc-α2,6 on the surface of the tracheal epithelium and alveoli. In all IAV-infected pigs, we found a low amount of IAV-positive cells in the trachea despite a high expression of the human receptor. Cumulatively, these findings suggest that optimal IAV replication involves a complex interplay between the viruses and their host receptors and that there might be other less clearly defined host factors that determine the site of replication.

## Highlights

• The avian influenza A virus receptor (SA-α2,3-Gal) isexpressed in the porcine nasal mucosa.
• The human influenza A virus receptors: Neu5Ac-α2,6 and Neu5Gc-α2,6 are equally expressed in the porcine trachea and alveoli.
• The human- and swine-adapted H1N1pdm09 and H3N2 viruses had a preferred tropism for the porcine bronchial and bronchiolar epithelial cells and were less prominent in the nose, trachea and alveoli.

## Graphical abstract

**Figure:**
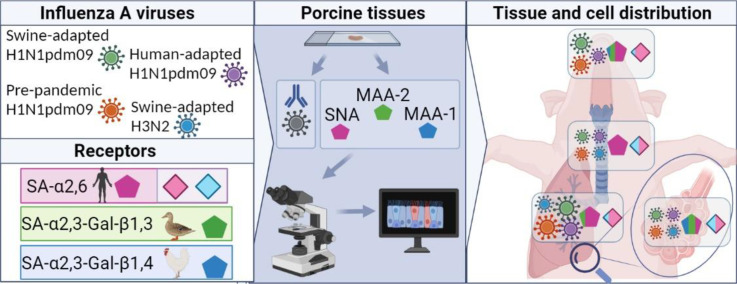

## 1 Introduction

Influenza A virus (IAV) is a negative, single-stranded, RNA virus. The natural hosts of IAV are waterfowls (Anseriformes) and shorebirds (Charadriiformes) (Yoon et al., 2014). Over time, IAV has crossed species barriers and is now the cause of respiratory disease in humans and a variety of mammals including pigs, horses, dogs, and seals (Daly and Kembou-Ringert, 2021; Van Den Brand et al., 2016; Yoon et al., 2014). IAV has two highly variable surface glycoproteins hemagglutinin (H) and neuraminidase (N) (Yoon et al., 2014). The combination of the H and N proteins defines the subtype of a given IAV. Currently, a wide variety of IAV subtypes with H1 to H16 and N1 to N9 have been isolated from birds (Yoon et al., 2014), whereas H1N1, H1N2, and H3N2 subtypes are enzootic in pigs (Gaëlle et al., 2014). The H1N1 and H3N2 subtypes constitute the present seasonal circulating subtypes in humans (Mook et al., 2020). H is important for the viral attachment on the host cell and facilitates viral entry, while N is important for penetrating the mucus layer of the respiratory tract and viral release (Dou, 2018). For all IAVs, membrane fusion and hence release of the viral genome complex into the cell cytoplasm require cleavage of the H into H1 and H2 subunits by host proteases (Garten et al., 2015).

**Fig. 1:**
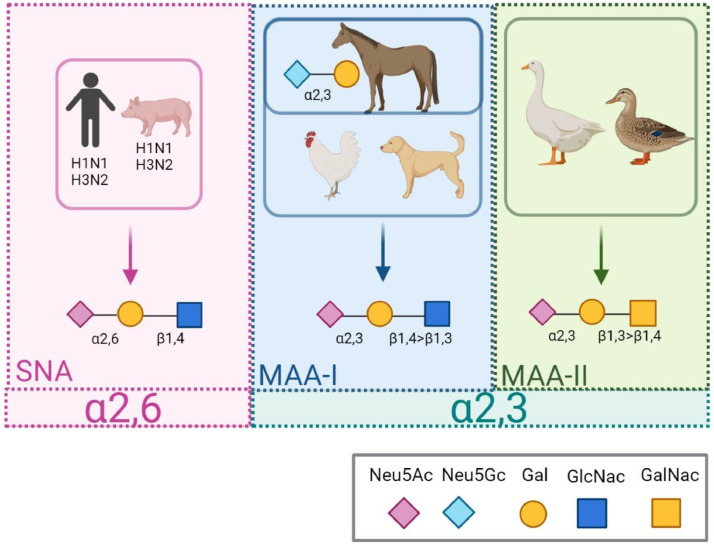
Illustration of the general receptor binding preferences of different host species of influenza A virus and the main binding affinity of Sambucus Nigra Lectin (SNA), Maackia amurensis Lectin I (MAA-I), and Maackia amurensis Lectin II (MAA-II) lectins. Neu5Ac= N-Acetylneuraminic acid. Neu5Gc= N-Glycolylneuraminic acid. Gal= galactose. GlcNac= N-acetylglucosamine. GalNac= N-acetylgalactosamine. Made in Biorender.com.

H binds to sialic acids (SA) terminally attached to glycans which facilitate endocytosis (Dou, 2018). In general, human and swine IAV isolates have a higher preference for SAs that are linked to galactose (Gal) in an α2,6 manner (SA-α2,6, referred to as human receptor), whereas avian isolates have a higher propensity for SAs that are linked to Gal in an α2,3 manner (SA-α2,3, referred to as avian receptor) (Bradley, 2011; Rogers and D´Souza, 1989; Sriwilaijaroen et al., 2018; Zhao and Pu, 2022). IAVs isolated from ducks generally prefer SA-α2,3-Gal that are linked to N-acetylglucosamine (GlcNac) in a β1,3 manner (SA-α2,3-Gal-β1,3-GlcNac referred to as duck receptor), whereas IAVs isolated from chickens generally prefer a β1,4 linkage to N-acetylgalactosamine (GalNac, SA-α2,3-Gal-β1,4-GalNac referred to as the chicken receptor) (Gambaryan et al., 2008, Gambaryan et al., 2018). Additionally, canine and equine IAVs also prefer the chicken receptor (Gambaryan et al., 2012; Wen et al., 2018). The general receptor binding preferences of IAVs isolated from selected animal species are illustrated in Fig. 1.

The distribution of the human receptor and avian receptors can be investigated by mass spectrometry, however, *in situ* methods are needed to investigate the level of expression in specific cell types (Varki and Varki, 2007). Sambucus Nigra Lectin (SNA) binds to the human receptor, while Maackia Amurensis Lectin I (MAA-I) binds to the avian receptor (Bojar, 2022; Büll, 2021; Gao et al., 2019; Narimatsu et al., 2019; Padler-Karavani et al., 2012), and more specifically to the chicken receptor (Gao et al., 2019; Narimatsu et al., 2019). Maackia Amurensis Lectin II (MAA-II) also binds to the avian receptor but with a higher preference for the duck receptor than the chicken receptor (Bojar, 2022; Büll, 2021; Padler-Karavani et al., 2012). In most of the previous studies (Eriksson et al., 2018; Trebbien et al., 2011; Nelli et al., 2010) pre-treatment was not performed prior to the lectin staining despite that pre-treatment of human tissues with citrate has been shown to increase the lectin staining significantly (Nicholls et al., 2007). Other studies were performed exclusively on porcine explant tissues (Van Poucke et al., 2010) or only assessed a part of the porcine respiratory tract (Chan, 2013). There are two major sialic acids, *i.e.* N-Acetylneuraminic acid (Neu5Ac) and N-Glycolylneuraminic acid (Neu5Gc) (Peri, 2018). Neu5Ac is expressed in humans and animals, whereas, Neu5Gc is present in horses and pigs (Spruit et al., 2021) and in different wildlife species including wild boars (Nemanichvili et al., 2022). Humans, domestic ferrets, birds, and other species do not express Neu5Gc due to a lack of the enzyme CMP-N-acetylneuraminic acid hydroxylase that converts Neu5Ac to Neu5Gc (Peri, 2018)*.* Equine IAVs are the only IAVs that have a higher preference for Neu5Gc than Neu5Ac (Broszeit, 2019; Gambaryan et al., 2012) however, a single amino acid substitution can improve the binding preference for Neu5Gc in IAV isolates from turkeys, chickens, and dogs (Spruit et al., 2022; Wen et al., 2018). Furthermore, a recent study showed that a change of binding affinity from Neu5Ac to Neu5Gc does not affect the fusion of IAV with the endosomal membrane because the fusion is only dependent on the H2 subunit (Chien, 2023). The difference in SA preference between avian IAV isolates (SA-α2,3) and the human or swine IAV isolates (SA-α2,6) constitute one of the most important species barriers between IAV in birds and mammalians (Bourret, 2018), but since human and swine IAV isolates have a similar preference for the SA-α2,6 receptor, this cannot explain why most swine-adapted IAVs have a decreased ability to infect humans. In contrast to humans, Neu5Gc has been reported to be extensively expressed in the cells of the porcine trachea (Sriwilaijaroen et al., 2011; Suzuki et al., 1997). The specific expression of Neu5Gc-α2,6 has not been investigated yet and it is still unclear if Neu5Gc can act as a functional receptor for IAV in pigs.

The main aims of this study were to investigate the *in situ* expression of Neu5Gc-α2,6 in the porcine nose, tracheal, and lung tissues by lectin histochemistry and to elucidate if the expression of this receptor coincided with the viral tropism of selected pig- and human-adapted subtypes and strains of IAV. Furthermore, the IAV receptor distribution (SA-α2,3 and SA-α2,6) in the porcine nose, the upper and lower part of the trachea, and lung tissues were evaluated using an optimized lectin protocol.

## 2 Materials and methods

### 2.1 Tissue and viruses

Commercial Danish Landrace control pigs (8–9 weeks of age) obtained from previous studies (Kristensen et al., 2023b; manuscript in preparation) were used to evaluate the distribution patterns of IAV receptors in the nose, upper and lower part of the trachea, and lung tissues. Furthermore, tissues from pigs (8–9 weeks of age) inoculated intranasally with different strains of IAV circulating in pigs (swine-adapted) or in humans (human-adapted) were included, too. These IAV-infected tissues were also obtained from previous studies, A/swine/Denmark/3974–2p4/2020 (swine-adapted H1N1pdm09), A/Denmark/238/2020 (human-adapted H1N1pdm09), A/Swine/Mexico/AVX-39/2012 (pre-pandemic H1N1pdm09) (Kristensen et al., 2023b; manuscript in preparation), A/swine/Denmark/14348–9/2003 (swine-adapted H3N2), A/Denmark/304/2020 (human-adapted H3N2) and a human-adapted strain isolated from pigs during an outbreak in a Danish herd A/swine/Denmark/S3974–2/2020 (hu/sw H3N2) (manuscript in preparation) were used to investigate potential differences between the tissue and cell tropisms of the different IAVs. The accession numbers of the strains investigated in this study are listed in Table S1.

### 2.2 Lectin histochemistry for natural lectins

Detection of SA-α2,6 was performed using biotinylated SNA (B-1305-2, Vector Laboratories, California, USA) and detection of SA-α2,3 was performed using biotinylated MAA-I (B-1315-2, Vector Laboratories) and biotinylated MAA-II (B-1265-1, Vector Laboratories). Two µm formalin-fixed and paraffin-embedded sections were deparaffinized. The sections were pre-treated by microwaving with 10 millimolar (mM) citrate buffer pH 6 for 10 min and rested in the buffer for 15 min. The sections were washed 3 times for 5 min with Tris-buffered saline (TBS) pH 7.6 with Tris hydrochloride, NaCl, CaCl_2_ and MgCl_2_ added (lectin TBS) as recommended by Brooks and Hall, 2012. This washing step was performed after each step described below. To block nonspecific binding, the sections were blocked with avidin for 15 min, biotin for 15 min, and Ultra V block for 5 min. Biotinylated SNA, MAA-I, and MAA-II diluted 1:5000, 1:4000, and 1:4000 in lectin TBS, respectively, were added to the sections overnight at 4 °C. Streptavidin-alkaline phosphatase (TS-060-AP, AH diagnostics, Aarhus, Denmark), diluted 1:100 in lectin TBS, was added for 10 min. The staining was developed by adding Vector Blue (SK-5300, Vector Laboratories) for 10 min, and hereafter the sections were washed with distilled water 3 times for 5 min. The sections were mounted with glycerol-gelatine. A negative control was performed as described above but without adding the lectins. The lectin staining was performed on porcine nasal (*N* = 2), upper trachea (*N* = 2), lower trachea (*N* = 1), and lung tissues (*N* = 2).

SNA exhibits binding affinity to both Neu5Ac-α2,6 and Neu5Gc-α2,6 (Broszeit, 2019; Padler-Karavani et al., 2012). To investigate the specific binding of SNA to Neu5Ac-α2,6 in porcine tissues, an anti-Neu5Gc antibody (Biolegend, San Diego, CA, USA, Cat. No: 146903), diluted 1:200 in lectin TBS, was incubated overnight at 4 °C as an additional pre-treatment step before conducting the SNA protocol described above. The Neu5Gc block was applied to the porcine nose (*N* = 1), upper trachea (*N* = 2), and lung tissues (*N* = 2).

### 2.3 Neuraminidase control

To ensure the specificity of the lectins, lung tissues were pre-treated with neuraminidase (sialidase) obtained from *Clostridium perfringens* (5 units, Roche #1158588600). The neuraminidase was diluted in 50 mM acetate buffer to a concentration of 1 U/ml and the pH value was adjusted to 5.0. Hereafter, 100 µl neuraminidase was added to the lung tissues overnight at 37 °C, and the SNA, MAA-I, and MAA-II staining were performed as described in Section 2.2. For MAA-I and MAA-II, the neuraminidase pre-treatment was also performed on the nasal mucosa.

### 2.4 Image analysis of the lectin histochemistry

Every tissue section and lung compartment (bronchi, bronchioles, respiratory bronchioles, and alveoli) was represented by four images each. The surface of lamina epithelialis was manually selected as a region of interest (ROI) and thresholds were adjusted in ImageJ according to the different lectins, tissue, and staining variability (Schneider, 2012). Positive staining was measured as the percentage of area that was above the threshold in each ROI and the staining from the neuraminidase controls was subtracted. Finally, the total percentage of the area (% area SNA +% area MAA-I +% area MAA-II) was calculated, and the distribution of each of the lectins: SNA, MAA-I, and MAA-II were calculated in percentages, see Table 1. Finally, the median values from the two pigs were reported.

**Table 1:** Showing the distribution of the known IAV-receptors in the porcine respiratory tract detected by three different lectins (SNA, MAA-I, MAA-II)*.

Tissue	Cell type	SA-α2,6 (SNA)	Neu5Ac-α2,6^1^	Neu5Gc-α2,6^2^	SA-α2,3-Gal-β1,4 (MAA-I)	SA-α2,3-Gal-β1,3 (MAA-II)
Nasal mucosa	Ciliated cells	62%	57%^3^	5%	18%	20%
	Glands and ducts	+	+	−	+	+
Upper trachea	Ciliated cells	100%	51%	49%	0	0
	Goblet cells	+	+	−	0	0
	Glands	+	0	+	0	0
Lower trachea	Ciliated cells	100%^4^	−4	−	0	0
	Goblet cells	+	−	−	0	0
	Glands	+	−	−	0	0
Bronchi	Ciliated cells	94%	85%	9%	0	6%
	Goblet cells	+	+	−	0	0
	Glands	+	+	−	0	0
Bronchioli	Bronchiolar epithelium	69%	62%	7%	0	31%
	Respiratory bronchiolar epithelium	55%	43%	12%	0	44%
Alveoli	Pneumocytes	19%	10%	9%	35%	46%
	Alveolar macrophages	+	+	−	0	0
Vessels	Endothelial cells	+	+	−	+	+

⁎ : +positive staining was observed but the amount was not semi-quantified. −staining was not assessed. 0: no staining observed. The distribution of the lectins was calculated as follows (an example for SNA): (SNA%)/(SNA% +MAA-I%+ MAA-II%) × 100.

1 Obtained by performing adding an anti-Neu5Gc antibody (blocking Neu5Gc) prior to the SNA staining.

2 Obtained by subtracting the percentage of Neu5Ac-α2,6 from the percentage of the SA-α2,6 (SNA) surface staining.

3 Only based on one pig.

4 Only based on one pig and no SNA staining with Neu5Gc block was performed.

### 2.5 IAV immunohistochemistry (IHC)

Two µm sections of formalin-fixed and paraffin-embedded blocks were deparaffinized and washed with TBS pH 7.6 two times for 5 min. This washing step was performed after each step described below. The sections were blocked for endogenous peroxidase with 3% H_2_O_2_ for 10 min and then pre-treated with 0.018 g proteinase (CAS number: 9014-01-1, Sigma Aldrich, Saint Louis, USA) in 100 ml TBS for 5 min. The sections were then blocked with Ultra V block (TL-125-HLJ, AH diagnostics, Tilst, Denmark) and then anti-influenza A (nucleoprotein ((NP)) antibody (HYB 340–05, SSI-antibodies, Copenhagen S, Denmark), diluted 1:100000 in 1% BSA/TBS, were added overnight for 4 °C. UltraVision ONE HRP-Polymer (TL-125-HLJ, AH diagnostics) was added for 30 min and the staining was developed by adding DAB substrate (957D-40 500, Cell Marque, California, 95677, United States) for 10 min. The sections were counterstained by Mayer's hematoxylin (AMPQ00254.5000, VWR, Pennsylvania, USA). An isotype control (IgG1, (X0931, Agilent, Santa Clara, California, USA)), diluted in 1% BSA/TBS to the same protein concentration as the anti-influenza A (NP) antibody was performed. There was a varying amount of tissue sections available for the different IAV-infected pigs and tissues, therefore, the numbers are summarized in Table 2.

**Table 2:** Showing the host tropism of different host-adapted IAV strains and subtypes*.

Tissue	Cell type	Swine-adapted H1N1pdm09		Human-adapted H1N1pdm09		Pre-pandemic H1N1pdm09		Swine-adapted H3N2	
		Positive/total	Score	Positive/total	Score	Positive/total	Score	Positive/total	Score
Nasal mucosa	Ciliated cells	4/4	5–15%	1/2	<5%	1/2	5–15%	0/4	0
	Glands		+		0		0		0
Upper and lower trachea	Ciliated cells	5/5	<5%	1/3	<5%	1/2	<5%	4/4	< 5%
	Goblet cells		0		0		0		0
	Glands		0		0		0		0
Bronchi	Ciliated cells	7/7	5–15%	3/8	5–15%	4/5	>15–25%	2/7	5–15%
	Goblet cells		0		+		+		0
	Glands		0		+		0		0
Bronchioli	Bronchiolar epithelium	7/8	>25–50%	3/8	>25–50%	6/6	>25–50%	3/7	5–15%
	Respiratory bronchiolar epithelium	7/8	>25–50%	3/8	5–15%	6/6	>15–25%	2/7	5–15%
Alveoli	Pneumocytes/ alveolar macrophages	7/8	<5%	3/8	<5%	6/6	<5%	3/7	<5%

⁎ The distribution of human-adapted H3N2 and hu/sw H3N2 are not presented because no IAV-positive cells were observed. 0: no positive staining observed, +: positive staining was observed but not quantified.

### 2.6 Image analysis of the IAV IHC

Each positive IAV section was scanned to a digital slide and lamina epithelialis was manually selected as ROI in QuPath (Bankhead, 2017). In lung tissues, at least two representative ROIs were selected for each lung compartment (bronchi, bronchioles, respiratory bronchioles, and alveoli). The tool “positive cell detection” was used to calculate the percentage of positive cells out of the total amount of cells in each ROI. The group median number of IAV-positive cells was calculated for each tissue or lung compartment.

### 2.7 Multiplex immunohistochemical staining of IAV and type II pneumocytes

Prosurfactant protein C (SP-C) is expressed in type II pneumocytes (Fujino et al., 2011) Therefore, to characterize IAV-positive type II pneumocytes a multiplex staining method was developed by using an anti-prosurfactant protein C antibody. From formalin-fixed and paraffin-embedded blocks, two µm sections were deparaffinized and washed with TBS, blocked with 3 % H_2_O_2,_ and pre-treated with proteinase as described in Section 2.5. The sections were also blocked with Ultravision protein block (TA060PBQ, Fischer Scientific, Hampton, New Hampshire, USA) for 5 min. The anti-SP-C antibody (ab40879, Abcam, Cambridge, UK), diluted 1:500 in 1 % BSA/TBS, was added to the sections overnight at 4 °C. A primary antibody enhancer (TL-060-PB, Fischer Scientific) was added for 20 min and then Ultravision large volume AP polymer (TL-060-AP, Fischer Scientific) was added for 30 min. The staining was visualized by adding Vector blue (SK-5300, Vector Laboratories) for 10 min. Anti-influenza A (NP) antibody, diluted 1:50000 in 1% BSA/TBS, was added to the sections overnight at 4 °C. UltraVision ONE HRP-Polymer was added for 30 min and the staining was developed by adding vector AEC (SK-4200, Vector Laboratories) for 10 min. The multiplex staining was performed on one lung section with the highest amount of IAV-positive pneumocytes from each IAV group (*n* = 1, *N* = 4). The isotype control for IgG1 was performed as described in Section 2.5. An IgG isotype control (X0903, Aglient), diluted in 1 % BSA/TBS to the same protein concentration as the anti-SP-C antibody, was used as a negative control.

## 3 Data availability

Raw measurements of the quantification of the IAV IHC and lectin histochemistry have been uploaded to Figshare.com with the DOI: 10.6084/m9.figshare.24829899.

## 4 Results

### 4.1 Receptor distribution

The negative control of the lectins showed only negligible nonspecific staining (Fig. S1A). After the neuraminidase treatment, all lectins showed significantly decreased staining on the surface of the respiratory epithelium in the porcine lung tissue (Figs. S1B–D & S3), indicating that the signal was specific, however, SNA stained the endothelial cells and MAA-II stained the subepithelial layer, indicating that this staining was nonspecific for both lectins.

**Fig. 2:**
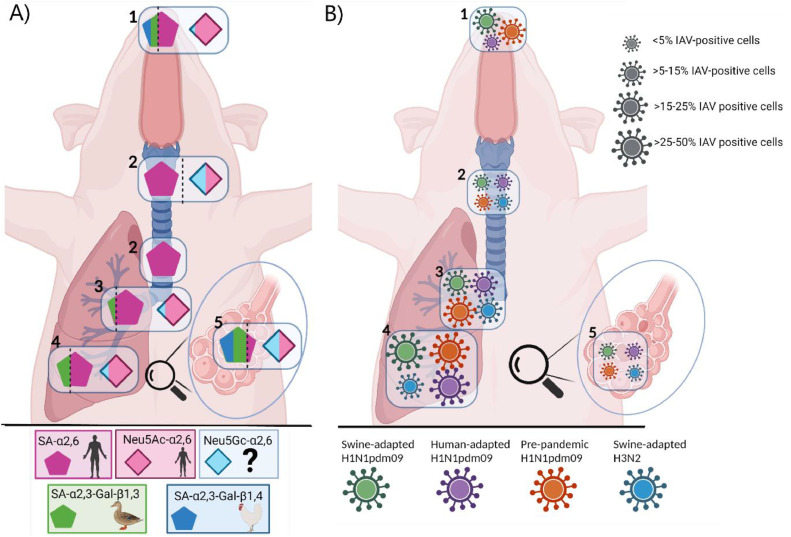
Comparison of the distribution of influenza A virus (IAV) receptors and the tissue tropism of different strains of IAV. (A) The distribution of IAV receptors was detected by lectin histochemistry and quantified in ImageJ: the human receptor (dark pink) was detected by Sambucus Nigra Lectin (SNA), the chicken receptor (dark blue) was detected by Maackia Amurensis Lectin I (MAA-I), and the duck receptor (green) was detected by Maackia Amurensis Lectin II (MAA-II) in the nasal mucosa (1), the upper and lower trachea (2), the bronchi (3), the bronchioles (4) and alveoli (5) of the porcine respiratory tract. The expression of Neu5Ac (light pink) and Neu5Gc (light blue) is also shown. B) The tissue tropism of swine-adapted H1N1pdm09 (green), human-adapted H1N1pdm09 (purple), pre-pandemic H1N1pdm09 (orange) and swine-adapted H3N2 (blue) isolates in the nasal mucosa (1), the trachea (2), the bronchi (3), the bronchioles (4) and alveoli (5) of the porcine respiratory tract was detected by IAV immunohistochemistry and semi-quantified in QuPath. Made in Biorender.com.

**Fig. 3:**
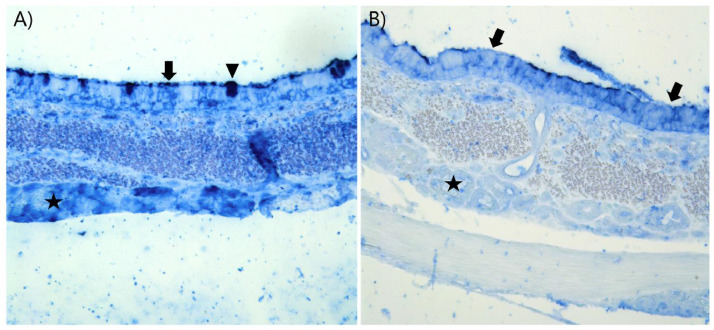
Reduced staining of Sambucus Nigra Lectin (SNA) after N-Glycolylneuraminic acid (Neu5Gc) pretreatment was observed in the porcine tracheal tissues. (A) Strong staining of SNA (dark blue) on the surface of the porcine tracheal epithelium (arrow), in goblet cells (arrowhead) and tracheal glands (star) investigated by SNA lectin histochemistry. (B) Reduced staining of SNA (dark blue) on the surface of the tracheal epithelium (arrows) and no staining of the tracheal glands (star) investigated by Neu5Gc block and subsequently SNA lectin histochemistry.

Using the SNA lectin, the human receptors (Neu5Ac and Neu5Gc) were detected on the surface of the respiratory epithelium throughout the porcine respiratory tract, however, less positive staining was present in the alveoli (Table 1, Figs. 2A & S2). Furthermore, the human receptors (SNA) was detected in the nasal glands and ducts, tracheal and bronchial goblet cells and glands, and alveolar macrophages (Table 1). To investigate if the signal of SNA was due to the binding of Neu5Ac and Neu5Gc, tissues were blocked with an anti-Neu5Gc antibody prior to the SNA staining. This antibody treatment allowed us to discriminate between the Neu5Ac and Neu5Gc expression. In the nasal mucosa, SNA stained 62% of the surface, whereas Neu5Ac-α2,6 consisted of 57% and Neu5Gc-α2,6 of 5%. (Table 1). In the upper part of the trachea, SNA stained 100% of the surface, whereas 51% consisted of Neu5Ac-α2,6 and 49% of Neu5Gc-α2,6 (Fig. 3). In the alveoli, the SNA surface staining consisted of 10% Neu5Ac-α2,6 and 9% Neu5Gc-α2,6. Overall, this showed that Neu5Gc-α2,6 only constituted a significant part of the SNA staining in the porcine trachea and alveoli (Table 1). The staining in lamina propria of nasal, tracheal, and bronchial tissues was markedly reduced after the Neu5Gc block (Fig. 3).

Both avian receptors (SA-α2,3-Gal-β1,3 and SA-α2,3-Gal-β1,4) were detected on the surface of the nasal epithelium (Table 1, Figs. 2A & S2). The chicken receptor (SA-α2,3-Gal-β1,4) was only found on the surface of the nasal mucosa (18% of the surface) and alveoli (35%), while the duck receptor (SA-α2,3-Gal-β1,3) was detected on the surface of the nasal mucosa (20%), the bronchi (6%), bronchioles (31%) and especially in the respiratory bronchioles (44%) and alveoli (46%) (Table 1, Figs. 2A & S2).

Overall these results showed that the human receptor was detected on the surface of the epithelium throughout the porcine respiratory tract, while the two avian receptors differed in distribution but both were expressed on the surface of the epithelium in the nasal mucosa.

### 4.2 Tropism of different IAV subtypes and strains

In all IAV-infected pigs, the highest number of IAV-positive cells was detected in the respiratory epithelium. All infected pigs had the highest number of IAV-positive cells in the bronchioles (Table 2, Fig. 2B). The swine-adapted H3N2 group also had the highest number of IAV-positive cells in the bronchi (Table 2, Fig. 2B). None of the pigs infected with swine-adapted H3N2 had IAV-positive cells in the nasal mucosa. Almost all of the infected pigs had the lowest number of IAV-positive cells in the trachea and alveoli, whereas pigs infected with the human-adapted H1N1pdm09 strain also had a low number of IAV-positive cells in the nasal mucosa (Table 2, Fig. 2B). A few numbers of IAV-positive cells were found in the nasal glands, goblet cells of the bronchi, and in the bronchial glands (Table 2). No IAV-positive cells were detected in the respiratory tissues of pigs infected with the human-adapted H3N2 isolates (human-adapted H3N2 and hu/swH3N2).

**Fig. 4:**
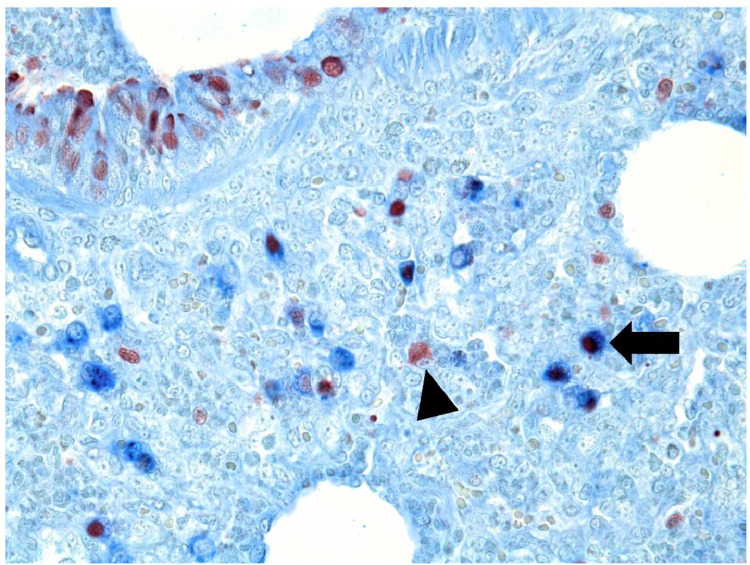
A pig infected with swine-adapted H1N1pdm09 showed influenza A virus (IAV) positive type II pneumocytes and leukocytes. The IAV-positive type II pneumocytes were detected with a multiplex immunohistochemically staining of IAV (red, arrowhead: IAV-positive leukocyte) and prosurfactant type C (type II pneumocytes, blue) revealing IAV-positive type II pneumocytes (red and blue, arrow).

In all pigs, IAV-positive leukocytes were detected, whereas IAV-positive type II pneumocytes were detected in pigs infected with swine-adapted H1N1pdm09, pre-pandemic H1N1pdm09, and swine-adapted H3N2 but not in pigs infected by the human-adapted H1N1pdm09 (Fig. 4). The isotype controls for the IAV (NP) and SP-C antibodies were both negative indicating that the multiplex staining of IAV and SP-C was specific (Fig. S4).

In summary, the highest number of IAV-positive cells were found in the porcine bronchi and bronchioles, whereas the lowest number was found in the nose, trachea, and alveoli.

## 5 Discussion

This is the first study that shows expression of the avian receptors (SA-α2,3) *in situ* on the surface of the epithelial cells in the porcine nasal mucosa, but the findings are consistent with previous findings in porcine respiratory explants (Van Poucke et al., 2010). The detections of both human and avian receptors in the porcine respiratory tract are comparable to findings in the human respiratory tract (Eriksson et al., 2018; Nicholls et al., 2007; Shinya and Ebina, 2006). Using lectins that specifically stain either the duck receptor (SA-α2,3-Gal-β1,3) or the chicken receptor (SA-α2,3-Gal-β1,4) we showed that these differed in distribution. The chicken receptor was expressed in the nasal mucosa and alveoli, whereas the duck receptor was present in all respiratory compartments, except in the trachea. These findings were overall in agreement with findings in nasal explants (Van Poucke et al., 2010) but disagreed with the findings of other studies (Eriksson et al., 2018; Nelli et al., 2010; Trebbien et al., 2011). These contrary findings could be explained by the lack of pre-treatment before performing the lectin histochemistry (Nicholls et al., 2007) or due to age differences between the pigs (Kimble et al., 2010; Nicholls et al., 2007).

We found an equal distribution of the chicken and duck receptors in the porcine nasal mucosa, which is a novel and interesting finding. Van Poucke et al. (2010) only found the duck receptor present in porcine nasal explants. This may be explained by a difference between *ex vivo* (nasal explant tissues) and *in situ* (porcine tissues). Previously the chicken receptor was reported in the human nasal mucosa (Nicholls et al., 2007; Shinya and Ebina, 2006). The finding of all receptors (human, chicken, duck) in the nasal epithelium of pigs further supports the hypothesis of pigs being a potential host for infection with IAVs from different species and sustains that pigs are candidates for being mixing vessels for new IAV strains (Hass et al., 2011; Webster et al., 1992; Bourret, 2018).

Our results documented that MAA-I and MAA-II have an SA-specific binding due to the significantly decreased staining after the neuraminidase pre-treatment even though some studies report that MAA-I and MAA-II also bind to Gal with no SA attached (Bojar, 2022; Büll, 2021; Gao et al., 2019; Narimatsu et al., 2019; Padler-Karavani et al., 2012).

In this study, we showed an equal *in situ* expression of Neu5Ac-α2,6 and Neu5Gc-α2,6 on the surface of the tracheal epithelium for the first time (Table 1). These findings are consistent with a previous study showing an equal expression of Neu5Ac and Neu5Gc in the porcine trachea using High Performance Liquid Chromatography (HPLC) (Suzuki et al., 1997).

Interestingly, it was not possible to detect any IAV-positive cells in pigs infected with the human-adapted H3N2’s (human-adapted H3N2 and hu/sw H3N2). Human H3N2’s isolated after 2008 have a receptor preference for the human receptor with long N-Acetyllactosamine *(*LacNac) repeats and this is believed to be a part of the host adaptation because humans express long LacNac repeats in the respiratory tract (Peng, 2017, Walther, 2013) except in the alveoli (Sriwilaijaroen et al., 2018). Contrary, the porcine respiratory tract expresses mainly a single LacNac chain (Chan, 2013) and this might explain the lack of IAV-positive cells in the pigs infected with the human H3N2 isolates. Another possible explanation could be that the viral load in these groups was too small to be detected by IAV IHC. Furthermore, no IAV-positive cells were found in the nose of pigs infected with the swine-adapted H3N2, which could be because IAV induces necrotic rhinitis with desquamation of epithelial cells (Kristensen et al., 2023b), and therefore, no IAV-positive cells were detected in the nasal mucosa of these pigs. These pigs may still be shedding the virus due to the replication of the virus in other respiratory compartments (Walther et al., 2013; Wong et al., 2019).

The porcine tracheal tissues were among the tissues with the lowest amount of IAV-positive cells, despite being the only tissue exclusively expressing the human receptor., Interestingly, the trachea was also the tissue with the highest expression of the Neu5Gc-α2,6 receptor. Neu5Gc has previously been shown to act as a decoy receptor (Takahashi et al., 2014) and therefore it is tempting to speculate that the binding of the viruses to this receptor prevented or decreased the infection of the tracheal epithelial cells. Another explanation for the low viral load in the trachea could also be the limited expression of voltage-gated calcium channels (VDCCs) in this tissue since we and others previously have found that VDCCs are important for the internalization process of IAV in pigs (Kristensen et al., 2023a, Fujioka et al., 2018). A novel study described that a high density of high-binding receptors and an additional presence of low-binding receptors increase the viral binding of recombinant human H1N1, H3N2, and avian H5N1 viruses compared to the presence of only a high density of high-binding receptors (Liu et al., 2022). Thus, a rather controversial explanation for the limited infection of the tracheal epithelial cells could be that co-expression of both the human and avian receptors enhances IAVs ability to infect the cells. Additional experiments are needed to confirm these findings and to investigate the underlying mechanism.

Some differences between viral loads observed in this study (using IAV IHC) and our previous study (using qPCR) were observed (Kristensen et al., 2023b). A possible explanation for this discrepancy is that PCR also measures the presence of IAV in the mucus, whereas the amount of mucus is decreased in IAV IHC due to the decreased mucus preservation when tissues are fixed in 10 % neutral buffered formalin (Blick, 2019). Furthermore, IHC detects proteins whereas qPCR targets RNA.

This study provides new insight into the IAV host receptors. We documented the expression of both avian receptors in the porcine nasal mucosa for the first time. Furthermore, we found an equal expression of Neu5Ac-α2,6 and Neu5Gc-α2,6 in the porcine trachea together with a low viral load despite a high expression of the human receptor. Additional research is needed to better understand the IAV tropism and host receptor relationship. Furthermore, additional work on IAV host receptors is required to elucidate why no IAV-positive cells were detected in pigs infected with human H3N2 isolates.

## CRediT authorship contribution statement

**Charlotte Kristensen:** Conceptualization, Methodology, Validation, Formal analysis, Writing – original draft, Visualization. **Lars E. Larsen:** Conceptualization, Formal analysis, Methodology, Project administration, Funding acquisition, Writing – review & editing. **Ramona Trebbien:** Methodology, Formal analysis, Writing – review & editing. **Henrik E. Jensen:** Validation, Methodology, Formal analysis, Writing – review & editing.

## Declaration of Competing Interest

The authors declare that they have no known competing financial interests or personal relationships that could have appeared to influence the work reported in this paper.

## Data Availability

We have made a data availability statement with a link to access raw measurements data.

## Appendix Supplementary materials

Image, application 1
