# Supplementary material for: The avian influenza A virus receptor SA-α2,3-Gal is expressed in the porcine nasal mucosa sustaining the pig as a mixing vessel for new influenza viruses
Source: Virus Res. 2024 Jan 3;340:199304. doi: 10.1016/j.virusres.2023.199304 (PMC10793167; doi:10.1016/j.virusres.2023.199304)
Supplement: Supplementary file 1 [file mmc1.docx]

**Supplementary files – the avian influenza A virus receptor SA-α2,3-Gal is expressed in the porcine nasal mucosa sustaining the pig as a mixing vessel for new influenza viruses**


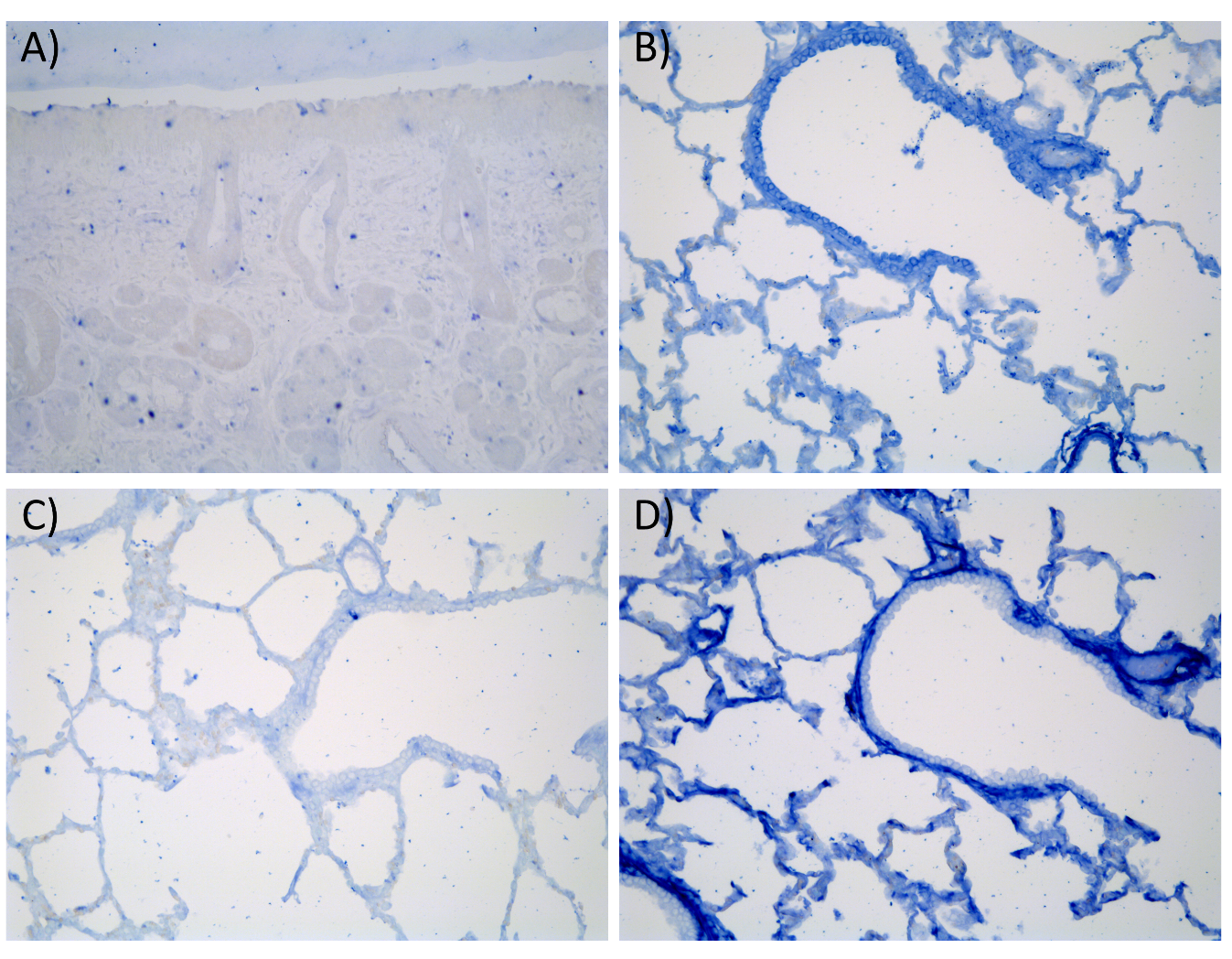


**Figure S1. Negligible unspecific staining observed by the negative control of the lectin histochemistry and neuraminidase controls of the lectins.** A) Negative control of the lectin histochemistry performed on the porcine nasal mucosa. B) Sambucus Nigra Lectin (SNA) lectin histochemistry of neuraminiadase pre-treated porcine lung tissues. C) Maackia amurensis Lectin I (MAA-I) lectin histochemistry of neuraminiadase pre-treated porcine lung tissues. D) Maackia amurensis Lectin II (MAA-II) lectin histochemistry of neuraminiadase pre-treated porcine lung tissues.


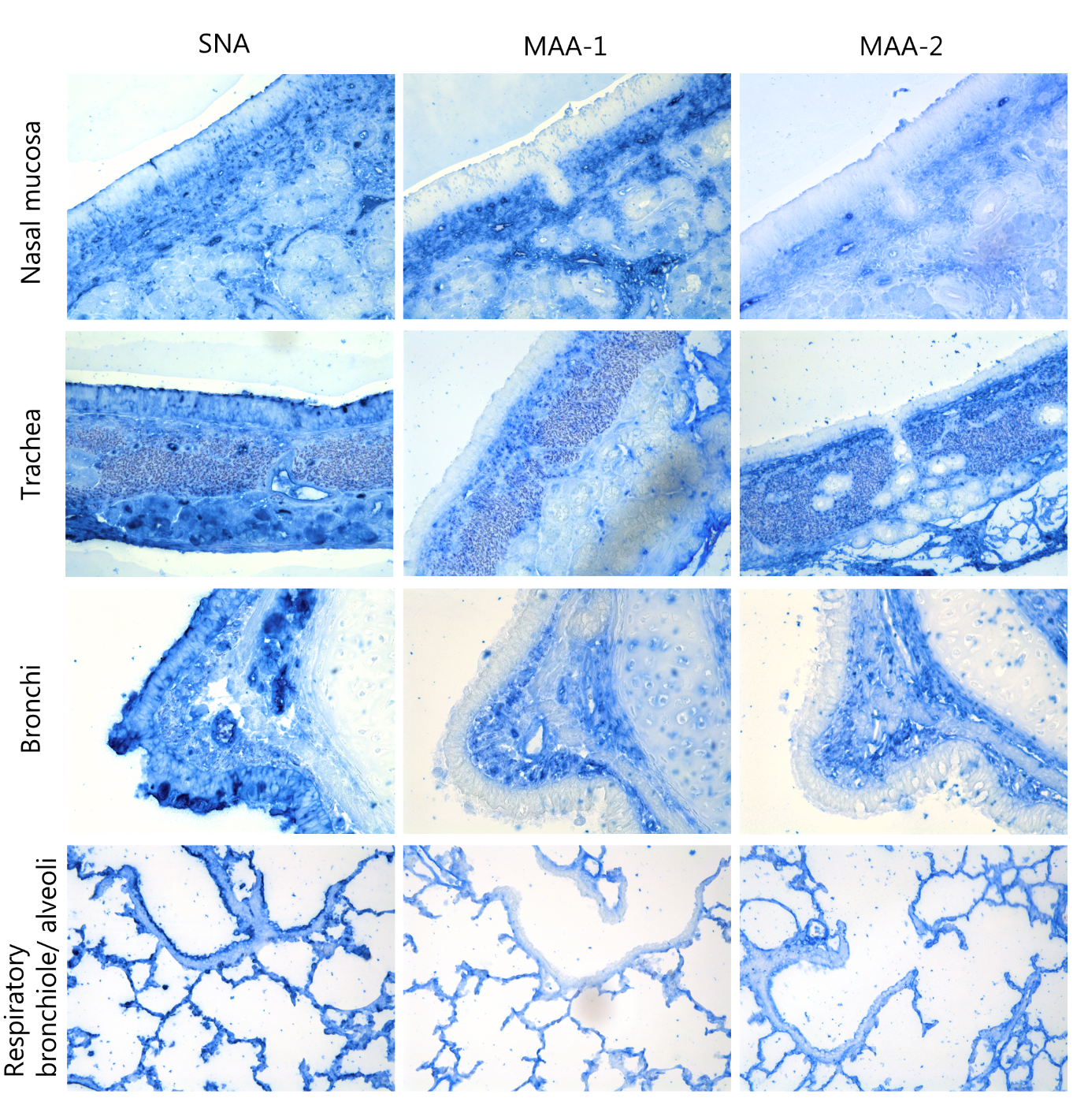


**Figure S2. The expression of Sambucus Nigra Lectin (SNA, human IAV receptor), Maackia amurensis Lectin I (MAA-I, chicken IAV receptor), and Maackia amurensis Lectin II (MAA-II, duck IAV receptor) differed in the porcine respiratory tract.** SNA was present throughout the respiratory tract while MAA-I was present in the nasal mucosa and alveoli, and MAA-II was expressed throughout the respiratory tract but absent in the porcine trachea.


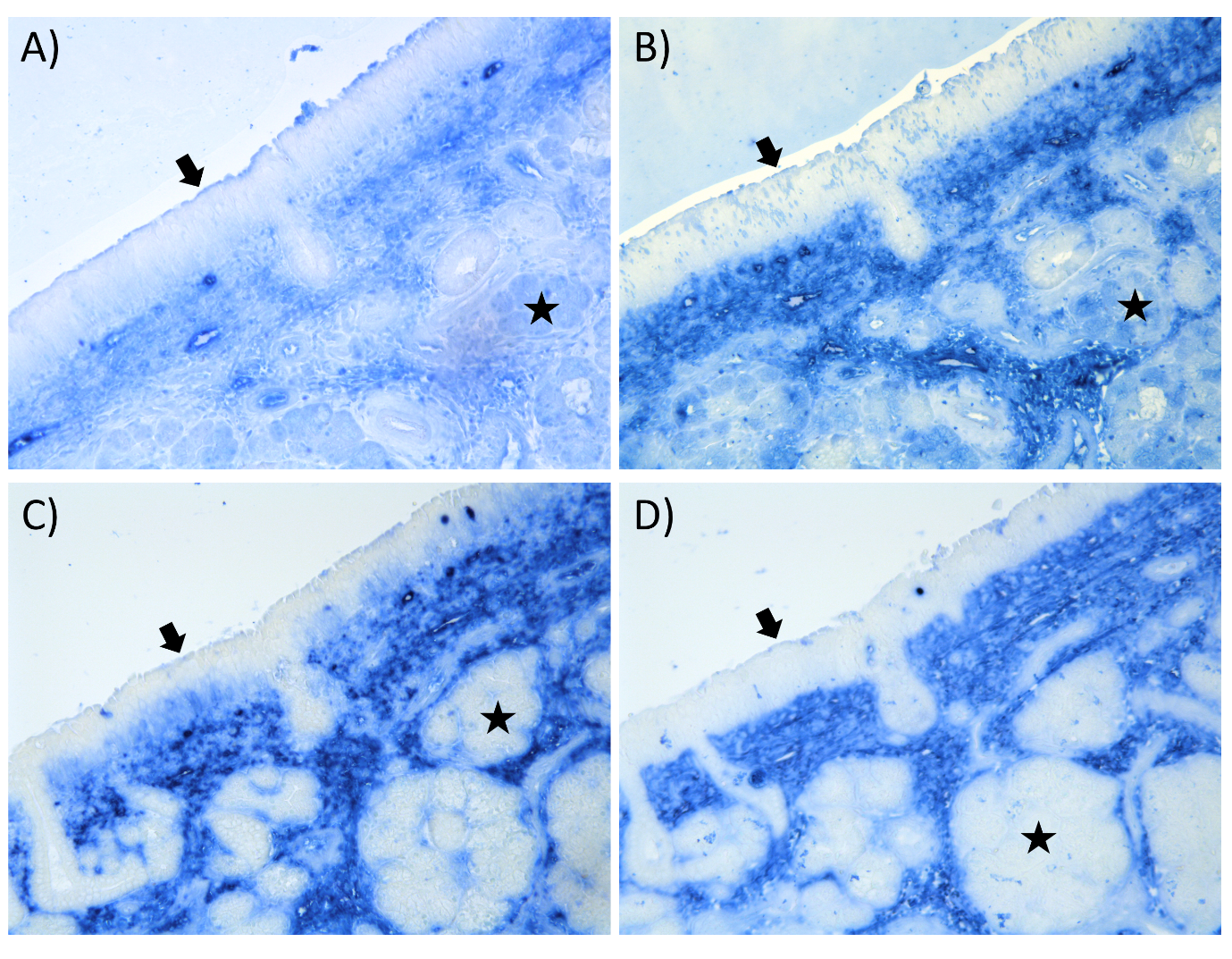
**Figure S3. Significantly reduced staining of Maackia amurensis Lectin I (MAA-I) and Maackia amurensis Lectin II (MAA-II) after neuraminidase pre-treatment.** A) MAA-II staining on the surface of the nasal mucosa epithelium (arrow) and variable staining of the nasal glands (star). B) MAA-1 staining on the surface of the nasal mucosa epithelium (arrow) and variable staining of the nasal glands (star). C) Neuraminidase pre-treatment and reduced staining of MAA-2 on the surface epithelium of the nasal mucosa (arrow) and no staining of the glands (star). D) Neuraminidase pre-treatment and reduced staining of MAA-1 on the surface epithelium of the nasal mucosa (arrow) and no staining of the glands (star).


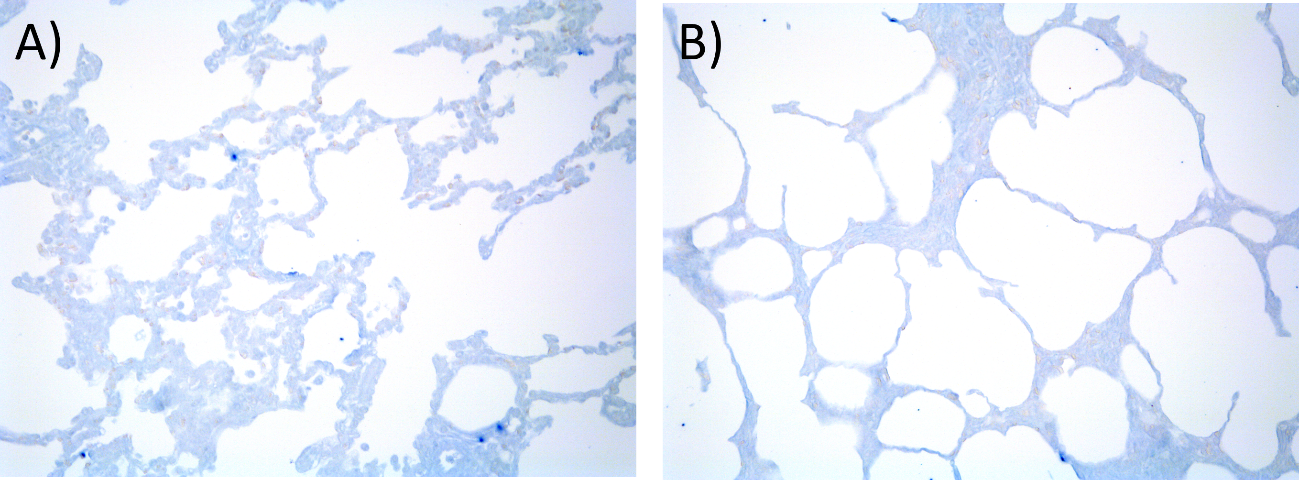


**Figure S4. No unspecific binding was observed with the isotype controls (IgG1, IgG) in the porcine lung tissues.** A) The isotype control (IgG1) for the influenza a virus immunohistochemistry staining of the porcine lung tissue. B) The isotype control (IgG) for the prosurfactant protein C immunohistochemistry staining of porcine lung tissue.

**Table S1.**

We gratefully acknowledge the authors, originating and submitting laboratories of the sequences from NCBI’s Influenza Virus Resource at GenBank on which this research is based. The list is detailed below.

| **Strain** | **Gene** | **Accession number** | **Authors** | **Paper reference** |
| --- | --- | --- | --- | --- |
| A/swine/Denmark/2017_10298/4_4p1/2017 | PB2 | MT666901 | Ryt-Hansen, P.; Krog, J.S.; Breum, S.O.; Hjulsager, C.K.; Pedersen, A.G.; Trebbien, R.; Larsen, L.E. | Swine-adapted H1N1pdm09 |
|  | PB1 | MT666902 |  |  |
|  | PA | MT666903 |  |  |
|  | HA | MT666904 |  |  |
|  | NP | MT666905 |  |  |
|  | NA | MT666906 |  |  |
|  | M | MT666907 |  |  |
|  | NS | MT666908 |  |  |
| A/Denmark/238/2020 | PB2 | OQ062647 | Kristensen, C., Laybourn, H.A., Crumpton, J-C., Martiny, K., Webb, A., Ryt-Hansen, P., Trebbien, R., Jensen, H.E., Nissen, J.N., Skovgaard, K., Webby, R.J., Larsen, L.E. | Human-adapted H1N1pdm09 |
|  | PB1 | OQ062648 |  |  |
|  | PA | OQ062649 |  |  |
|  | HA | OQ062650 |  |  |
|  | NP | OQ062651 |  |  |
|  | NA | OQ062652 |  |  |
|  | M | OQ062653 |  |  |
|  | NS | OQ062654 |  |  |
| A/swine/Mexico/AVX39/2012 | PB2 | KU976900 | Mena, I., Nelson, M.I., Quezada-Monroy, F., Dutta, J., Cortes-Fernandez, R., Lara-Puente, H.J., Castro-Peralta, F., Cunha, L., Trovao, N., Lozano-Dubernard, B., Rambaut, A., van Bakel, H. and Garcia-Sastre, A. | Pre-pandemic H1N1pdm09 |
|  | PB1 | KU976830 |  |  |
|  | PA | KU976786 |  |  |
|  | HA | KU976597 |  |  |
|  | NP | KU976910 |  |  |
|  | NA | KU976834 |  |  |
|  | M | KU976520 |  |  |
|  | NS | KU976514 |  |  |
| A/swine/Denmark/14348-9/2003 | PB2 | KC900241.1 | Trebbien, R., Bragstad, K., Larsen, L.E., Nielsen, J., Botner, A., Heegaard, P.M., Fomsgaard, A., Viuff, B. and Hjulsager, C.K. 2013 | Swine-adapted H3N2 |
|  | PB1 | KC900242.1 |  |  |
|  | PA | KC900243.1 |  |  |
|  | HA | KC900244.1 |  |  |
|  | NP | KC900245.1 |  |  |
|  | NA | KC900246.1 |  |  |
|  | M | KC900247.1 |  |  |
|  | NS | KC900248.1 |  |  |

Table S1 continued.

| **Strain** | **Gene** | **Accession number** | **Authors** | **Paper reference** |
| --- | --- | --- | --- | --- |
| A/Denmark/304/2020 | PB2 | OR994898 | Kristensen, C., Larsen, L.E., Trebbien, R., Jensen, H. E. | Human-adapted H3N2 |
|  | PB1 | OR994899 |  |  |
|  | PA | OR994900 |  |  |
|  | HA | OR994901 |  |  |
|  | NP | OR994902 |  |  |
|  | NA | OR994903 |  |  |
|  | M | OR994904 |  |  |
|  | NS | OR994905 |  |  |
| A/swine/Denmark/S3974-2/2020 | PB2 | OR994906 | Kristensen, C., Larsen, L.E., Trebbien, R., Jensen, H. E. | Hu/sw H3N2 |
|  | PB1 | OR994907 |  |  |
|  | PA | OR994908 |  |  |
|  | HA | OR994909 |  |  |
|  | NP | OR994910 |  |  |
|  | NA | OR994911 |  |  |
|  | M | OR994912 |  |  |
|  | NS | OR994913 |  |  |
